# Comparative transcriptome analysis of galls from four different host plants suggests the molecular mechanism of gall development

**DOI:** 10.1371/journal.pone.0223686

**Published:** 2019-10-24

**Authors:** Seiji Takeda, Makiko Yoza, Taisuke Amano, Issei Ohshima, Tomoko Hirano, Masa H. Sato, Tomoaki Sakamoto, Seisuke Kimura

**Affiliations:** 1 Graduate School of Life and Environmental Sciences, Kyoto Prefectural University, Kyoto, Japan; 2 Biotechnology Research Department, Kyoto Prefectural Agriculture Forestry and Fisheries Technology Center, Seika, Kyoto, Japan; 3 Department of Bioresource and Environmental Sciences, Faculty of Life Sciences, Kyoto Sangyo University, Kyoto, Japan; 4 Department of Industrial Life Sciences, Faculty of Life Sciences, Kyoto Sangyo University, Kyoto, Japan; 5 Center for Ecological Evolutionary Developmental Biology, Kyoto Sangyo University, Kyoto, Japan; Hainan University, CHINA

## Abstract

Galls are plant structures generated by gall–inducing organisms including insects, nematodes, fungi, bacteria and viruses. Those made by insects generally consist of inner callus–like cells surrounded by lignified hard cells, supplying both nutrients and protection to the gall insects living inside. This indicates that gall insects hijack developmental processes in host plants to generate tissues for their own use. Although galls are morphologically diverse, the molecular mechanism for their development remains poorly understood. To identify genes involved in gall development, we performed RNA–sequencing based transcriptome analysis for leaf galls. We examined the young and mature galls of *Glochidion obovatum* (Phyllanthaceae), induced by the micromoth *Caloptilia cecidophora* (Lepidoptera: Gracillariidae), the leaf gall from *Eurya japonica* (Pentaphylacaceae) induced by *Borboryctis euryae* (Lepidoptera: Gracillariidae), and the strawberry-shaped leaf gall from *Artemisia montana* (Asteraceae) induced by gall midge *Rhopalomyia yomogicola* (Oligotrophini: Cecidomyiidae). Gene ontology (GO) analyses suggested that genes related to developmental processes are up–regulated, whereas ones related to photosynthesis are down–regulated in these three galls. Comparison of transcripts in these three galls together with the gall on leaves of *Rhus javanica* (Anacardiaceae), induced by the aphid *Schlechtendalia chinensis* (Hemiptera: Aphidoidea), suggested 38 genes commonly up–regulated in galls from different plant species. GO analysis showed that peptide biosynthesis and metabolism are commonly involved in the four different galls. Our results suggest that gall development involves common processes across gall inducers and plant taxa, providing an initial step towards understanding how they manipulate host plant developmental systems.

## Introduction

Plants are not only food sources but also living microenvironments for other organisms. Plant galls are generated by insects, nematodes, fungi, bacteria, and viruses, among which, galls created by insects vary widely in terms of their shapes and colors. The estimated number of gall insect species ranges from 21,000 to 211,000 [1–2], and the structure of these galls is generally different from those of plant organs that develop normally, indicating that gall insects manipulate the plant developmental system and build a convenient structure for themselves [1].

Insect galls are induced by a wide range of species including flies, beetles, Hemiptera, wasps, midges, micromoths, and aphids. There is empirical evidence that effectors from insects, including phytohormones (auxin, cytokinin, and abscisic acids) and proteins are involved in gall generation [3–6]. Studies of green–island symptoms suggest that cytokinin supplied by insects to plants is synthesized by symbiont bacteria [7–8]. In some galls, initiation is stimulated by female oviposition [9]. This suggests that secretion from insects stimulate plant cell differentiation to generate the gall structure, although the molecular mechanism for gall initiation and development still remains unclear.

Gall development can be divided into the following processes: (1) secretion of signaling molecules from insects, (2) perception of the signals by plants, (3) plant cell regeneration and differentiation, and (4) organization of gall tissue. During these processes, insects need to suppress the plant’s defense responses [6]. Although many studies have described the gall structure and features, galls development seems to be a complex pathway, such that the molecular mechanism of gall development still remains unclear, due to wide variation in gall and host plant species. Recent progress in next generation sequencing (NGS) has allowed us to outline the biological processes in many organisms. Transcriptome analyses in several galls have been reported recently. For example, the gall transcriptome of *Metrosideros polymorpha*, induced by psyllid (Hemiptera), suggested the involvement of auxin response in the gall [10]. The horned galls of *Rhus chinensis* and *Rhus javanica* accumulate high amounts of tannins that make up to 60–70% of its total dry weight, protecting them from herbivory. Transcriptomes of both host plants and gall aphids have helped elucidate the molecular mechanisms of tannin biosynthesis and aphid reproduction, respectively [11–12]. Another example is the gall of wild grapevine (*Vitis riparia*) generated by phylloxera (*Daktulosphaira vitifoliae*), suggesting that pathways of floral organ development and procambium differentiation are involved in gall development [13]. These reports propose the molecular mechanism of interaction between gall insects and host plants, although, the gall structure varies widely making it difficult to identify the fundamental processes of gall development.

**Fig 1:**
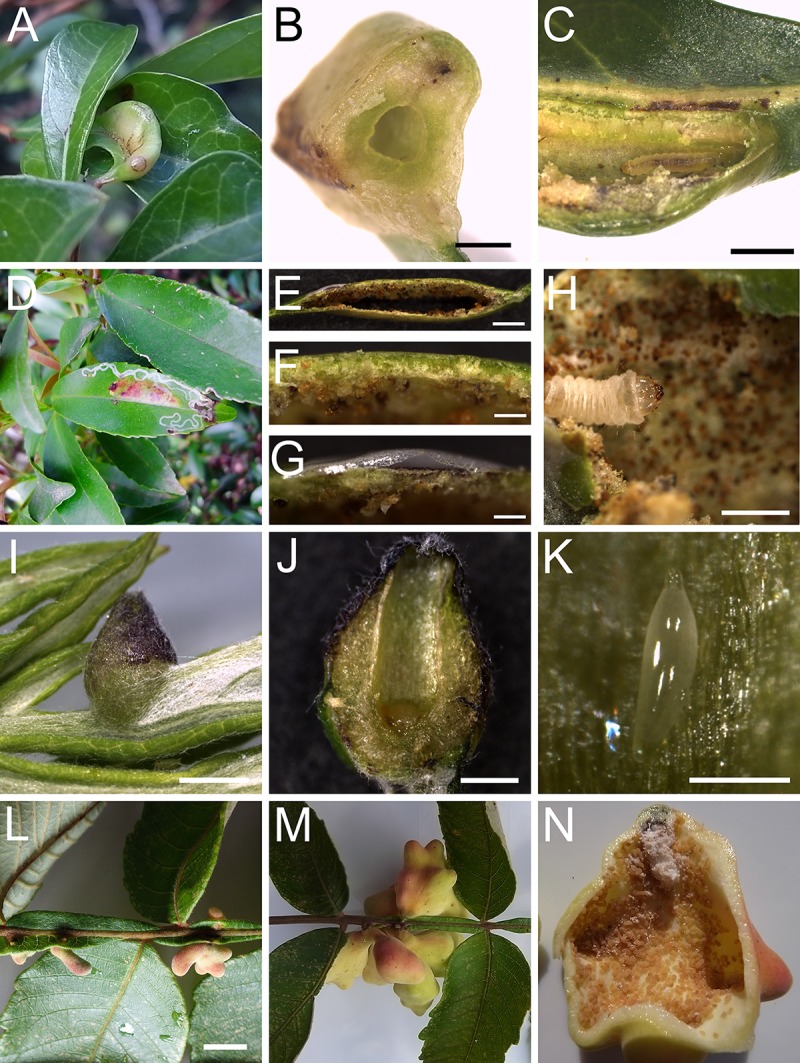
Galls used in this study. (A–C) The gall of *G*. *obovatum*. (A) The gall generated on a leaf. (B) Transverse section of the gall. (C) Longitudinal section of the gall, showing larva inside. (D–H) Gall of *E*. *japonica*. (D) Leaf showing the trace of leaf miner (white line) and the gall in the middle of the leaf. (E) Cross sections of the gall. Notably, this gall has rather thin layers compared to the other galls. (F) Upper part of the gall section of (E), showing thin layer of cells. (G) Cross section of trace of leaf miner in (E), showing the detached cuticle layer. (H) The larva inside the gall. (I–K) Galls of *A*. *montana*. (I) Intact gall on the leaf. (J) Longitudinal section of the gall. (K) Egg inside the gall. (L–N) Galls of *R*. *javanica*. (L) Early stage galls developing on the winged rachides. (M) Later stage galls. (N) Transverse section of the gall, showing many aphids living inside. Scale bars: B, E, H, J, 1 mm; C and I, 2 mm; F, G, K, 0.2 mm; L, 10 mm.

To understand the molecular mechanism of gall development, we performed RNA–sequencing–based transcriptome analyses for leaf galls from four different plant species. The leaf gall of *Glochidion obovatum* (Phyllanthaceae) (kankonoki–ha–fukure–fushi in Japanese, meaning swollen leaf gall of *G*. *obovatum*) is induced by the micromoth *Caloptilia cecidophora* (Lepidoptera: Gracillariidae), and develops into swollen and hard structures (Fig 1A–1C). The larva of this micromoth is the leaf miner up to the second instar, taking nutrients from leaf epidermal cells. After the third instar, it moves inside the leaves and generates a gall within leaf tissue [14]. Leaf gall of *Eurya japonica* (Pentaphylacaceae) (called hisakaki–ha–fukure–fushi in Japanese, meaning swollen leaf gall of *E*. *japonica*) is generated by another micromoth *Borboryctis euryae* (Lepidoptera: Gracillariidae), with a structure thinner than that of the gall of *G*. *obovatum* (Fig 1D–1H). This larva is also the leaf miner at an early stage, and later transforms to galling larva [15].

Together with these micromoth–induced galls, we selected the strawberry–shaped gall on leaves of *Artemisia montana* (Asteraceae), called yomogi–ha-eboshi–fushi (meaning *A*. *montana* hat–shaped gall on leaf, in Japanese), which is generated by a gall midge *Rhopalomyia yomogicola* (Oligotrophini: Cecidomyiidae) (Fig 1I–1K) [4]. Gene ontology (GO) analyses for transcripts in these three plant species suggested that development–related genes are upregulated in galls, whereas photosynthesis–related genes are downregulated. Comparison of transcripts in galls of these three species and another leaf gall on *Rhus javanica* (Fig 1L–1N), induced by the aphid *Schlechtendalia chinensis* (Hemiptera: Aphidoidea), suggested that 38 genes are commonly up–regulated in leaf galls from different plant species.

## Materials and methods

### Sample collection and microscopy

Galls on leaves of *G*. *obovatum* and *E*. *japonica* were originally collected from Tomogashima Island (Kada, Wakayama, Japan) and Kibogaoka Cultural Park (Yasu, Shiga, Japan), respectively, and both have been successfully reared in the laboratory [14,15]. For *G*. *obovatum*, the galls with the third instar larva were collected as young galls, and those with the fourth to fifth larva as mature galls. In both cases, the collected galls were cut in half and the larva removed. The intact leaves from the same tree were collected as control samples. For *E*. *japonica*, the gall with the fourth instar inside was collected, cut, and larva removed. Intact leaves from the same tree were collected as control samples. Galls and leaves of *A*. *montana* were collected from Kyoto Prefectural University, Seika campus (Seika, Kyoto, Japan). Gall and larva RNA were extracted to avoid physical stress by dissection, since the size of the gall was small. Collection, RNA extraction and RNA–sequencing of galls and leaves from *R*. *javanica* were performed by collaborators (Hirano and Sato, in preparation). All samples were frozen in liquid nitrogen and kept at -80°C until required for RNA extraction. Photos were taken with an S8AP0 stereomicroscope mounted with an EC3 digital camera (Leica, Germany).

### RNA extraction and RNA–sequence

In each plant species, three independent samples were used for RNA extraction. Total RNA was extracted from approximately 0.05 g of galls or leaves by two different methods. The RNA from *G*. *obovatum* young and mature leaves, and *E*. *japonica* leaves and galls were extracted using the Nucleospin RNA Plant and Fungi kit (Macherey–Nagel, Germany) following the manufacturer’s instruction. All other RNA extractions were performed using a modified protocol with the RNeasy Plant Mini Kit (QIAGEN, Germany) [16]. For RNA–seq analysis, 0.5 μg of the total RNA samples was used for library preparation after RNA integrity was confirmed by running samples on an Agilent RNA 6000 Nano Chip (Agilent Technologies, U. S. A). All libraries were prepared using Illumina TruSeq Stranded mRNA LT Sample kit according to the manufacturer’s instructions (Illumina, U. S. A). The pooled libraries were sequenced on an Illumina NextSeq500 sequencing platform, and single–end reads of 76 bp length were obtained. The reads from each species were assembled *de novo* into contigs using Trinity [17] with quality trimming of reads and strand specific assembly. The obtained reads were mapped to the *de novo* assembled RNA contigs using BWA (http://bio-bwa.sourceforge.net) [18]. The count data were subjected to a trimmed mean of M–value (TMM) normalization in EdgeR [19]. The transcript expression and digital gene expressions (DGEs) were defined using the EdgeR GLM approach [19], and genes with false discovery rates (FDRs) < 0.01, sum (total number of mapped reads) > 1, and log_2_FC > 1 (up–regulated) or log_2_FC < -1 (down–regulated) were classified as differentially expressed genes (DEGs), which were used for functional prediction by a BLASTX search against the Arabidopsis protein database (TAIR10). The gene number was estimated after the overlapped the Arabidopsis Gnome Initiative (AGI) number was eliminated. For GO analysis, we used PANTHER classification system through the TAIR database [20]. Accession numbers for the RNA–seq data are as follows: DRA008532 (*G*. *obovatum*), DRA008531 (*E*. *japonica*), and DRA008530 (*A*. *montana*), and one for *R*. *javanica* is described in another manuscript (Hirano and Sato, in preparation).

## Results and discussion

### Transcriptomes of galls from different plant species

**Fig 2:**
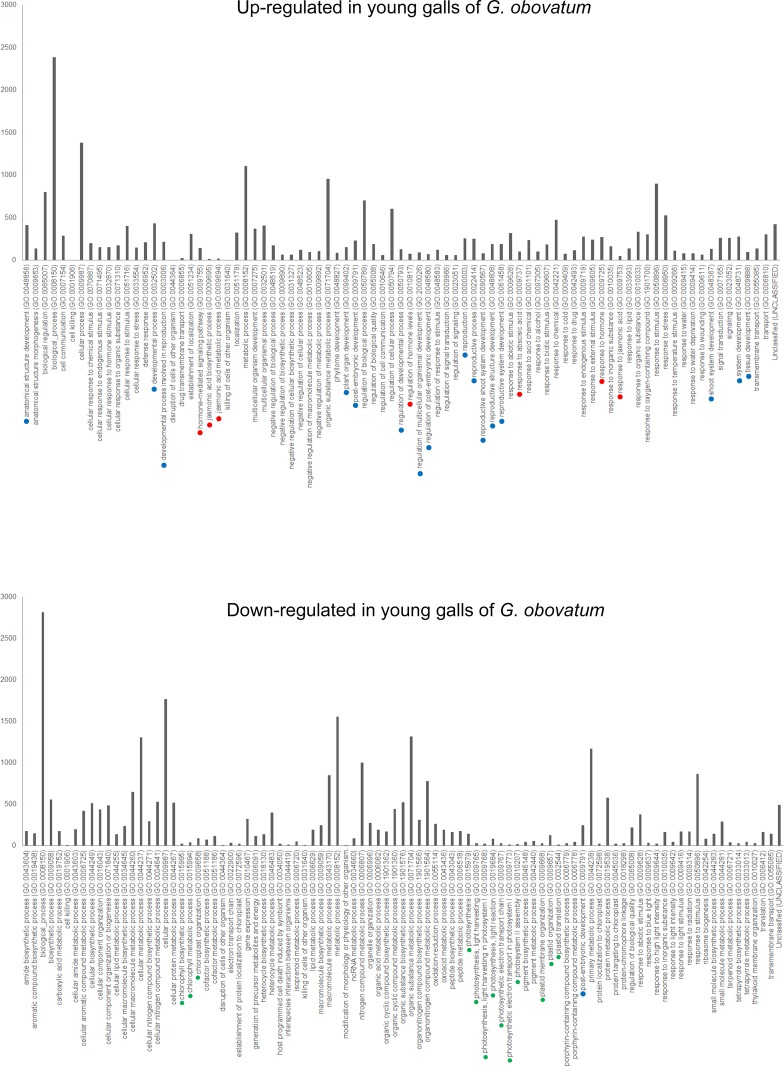
Gene ontology (GO) analysis (biological process) of young gall and leaf from *G*. *obovatum*. Colored dots indicate similar biological GO: blue, developmental process; red, phytohormone; and green, photosynthesis.

**Fig 3:**
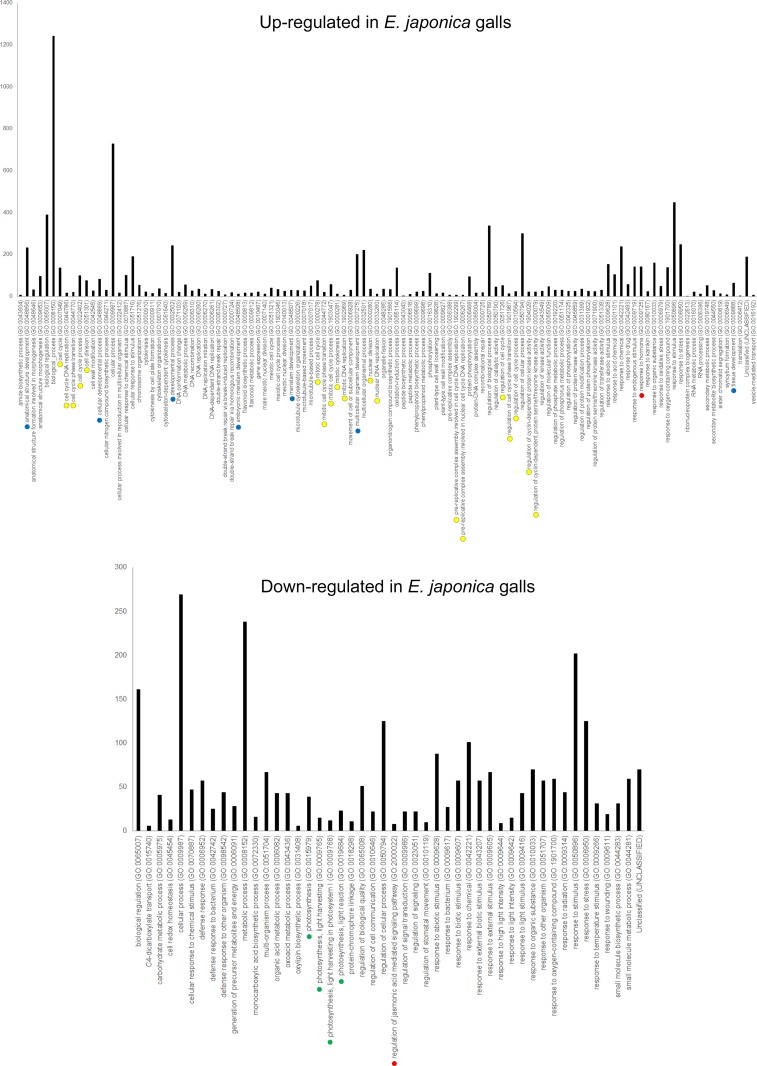
Gene ontology (GO) analysis (biological process) of gall and leaf from *E*. *japonica*. Colored dots indicate similar biological GO: blue, developmental process; red, phytohormone; yellow, cell cycle; and green, photosynthesis.

**Fig 4:**
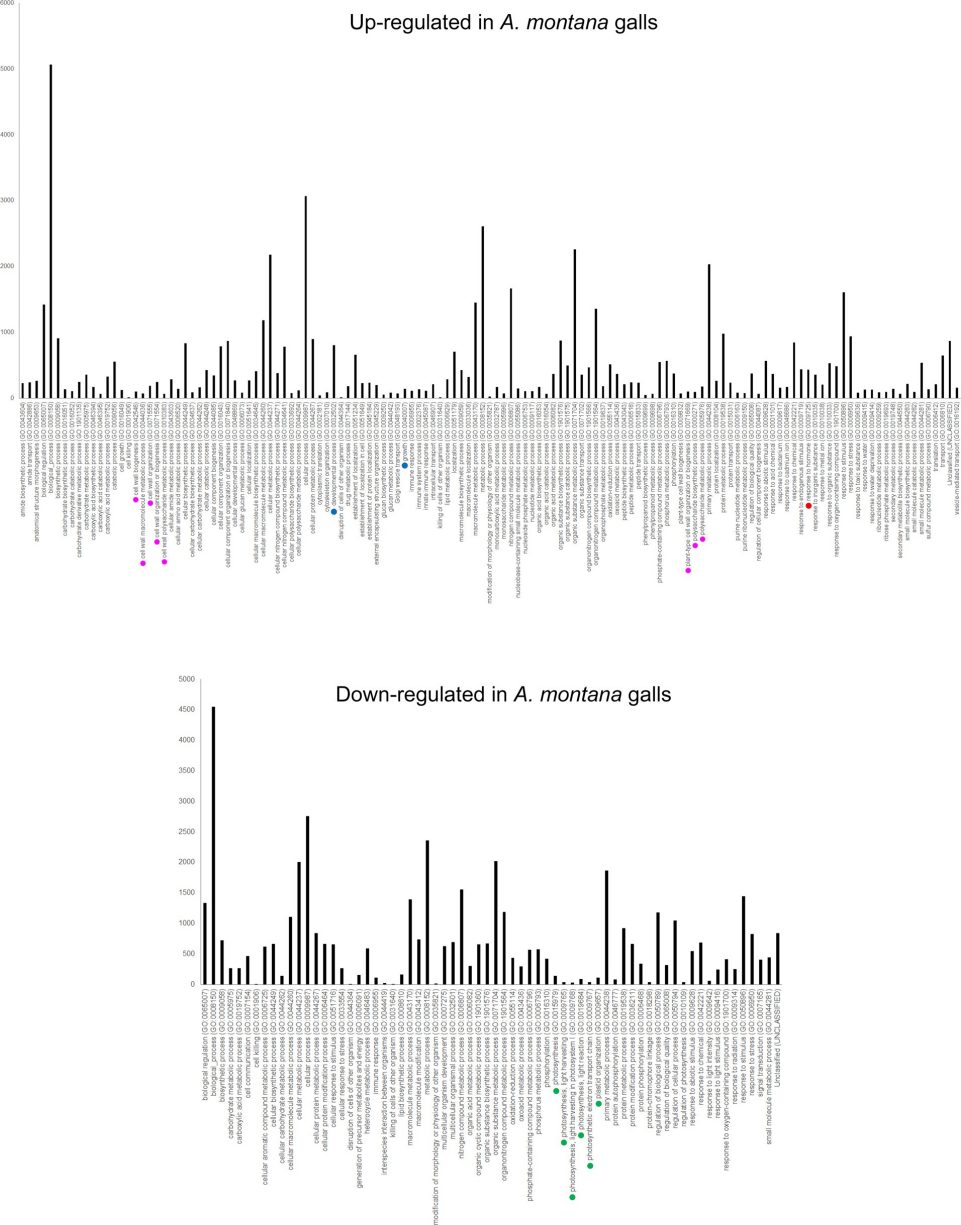
Gene ontology (GO) analysis (biological process) of gall and leaf from *A*. *montana*. Colored dots indicate similar biological GO: blue, developmental process; red, phytohormone; magenta, cell wall organization; and green, photosynthesis.

To elucidate the molecular mechanism of gall development, we isolated RNA from galls and leaves, followed by library construction and RNA–sequencing by NGS (S1 Table). For *G*. *obovatum* galls, we analyzed both young (inside larva at third instar) and mature galls (fourth to fifth instar). In both cases, genes related to developmental processes were up–regulated and photosynthesis–related genes were down–regulated in galls compared to those in leaves (Fig 2 and S1 Fig). The transcriptome of another micromoth–induced leaf gall on *E*. *japonica* suggested that genes related to development as well as cell cycle were up–regulated in galls (Fig 3). In leaf galls induced by the gall midge on *A*. *montana*, the genes related to developmental processes and cell wall organization were up–regulated (Fig 4). In these three galls, photosynthesis–related genes were down–regulated (Figs 2–4). These results suggest that leaf galls from different plant species commonly down–regulate the photosynthesis activity and express genes related to developmental process for gall morphogenesis. Notably, the three galls express different sets of genes, i.e., phytohormone–related genes in *G*. *obovatum*, cell cycle–related genes in *E*. *japonica*, and cell wall biosynthesis–related genes in *A*. *montana*. This difference may be one of the explanations for the unique shape of galls among different plant species.

### Four different galls expressed 38 common genes

**Fig 5:**
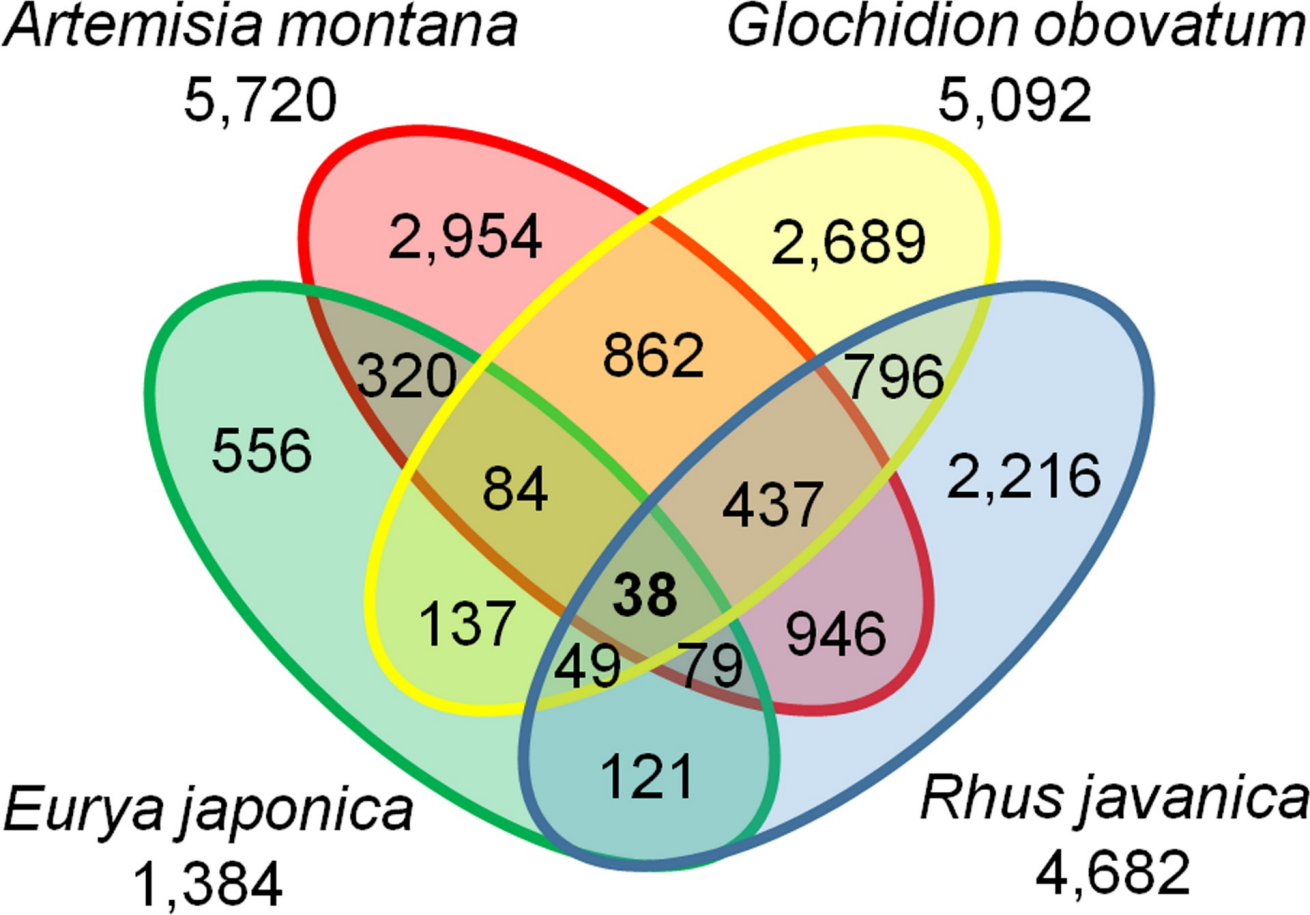
Venn diagram of transcriptome results for the 4 different galls. The number of genes that are upregulated more than twice than that in leaves is shown. Note that 38 genes are commonly expressed in the four galls.

The data from RNA–sequencing of *R*. *javanica* were added to our analysis (Hirano and Sato et al., in preparation). We selected gall–rich genes (genes expressed in galls more than twice that in leaves (see Materials and Methods), whose molecular functions were predicted by a homology search with BLASTX to the *Arabidopsis thaliana* protein database (TAIR10). For *G*. *obovatum*, data from young and mature galls and leaves were combined, and gall–rich genes compared to those in leaves were extracted. The AGI code corresponding to each gene sequence was compared among the four plant species. The gene number that was expressed more than twice in galls compared to that in leaves was as follows: *A*. *montana*, 5,720; *E*. *japonica*, 1,384; *G*. *obovatum*, 5,092; and *R*. *javanica*, 4,682 (Fig 5). With comparison among these datasets, we found that 38 genes are commonly expressed in four different galls (Fig 5 and Table 1). These 38 genes may include the master regulators for gall development in different plant species.

**Table 1:** Thirty–eight genes upregulated in 4 different galls.

Annotation	AGI (*Arabidopsis thaliana*)	logFC^a^				Putative molecular function	Putative biological function	References
		*A*. *montana*	*E*. *japonica*	*G*. *obovatum*	*R*. *javanica*			
ATBRCA1|breast cancer susceptibility 1	AT4G21070	8.38	4.07	1.35	7.24	DNA repair	cell cycle, DNA repair	[21–23]
FUSED Kinase family|Protein kinase family protein with ARM repeat domain	AT1G50240	4.74	2.94	4.54	9.68	protein kinase	cell division, cytokinesis	[24, 25]
Integrase-type DNA-binding superfamily protein ERF115	AT5G07310	3.60	8.82	5.15	2.51	transcription factor	cell cycle regulation	[26]
DOMINO1|Protein of unknown function (DUF3223)	AT5G62440	1.16	2.95	1.52	2.70	nuclear localization	cell division, nuclear size regulation	[27]
DUT1|DUTP-PYROPHOSPHATASE-LIKE1	AT3G46940	1.60	4.50	3.83	7.44	deoxyuridine triphosphatase (dUTPase)	DNA protection	[28]
AtTLP2,TLP2|tubby-like protein 2	AT2G18280	1.01	2.89	2.22	2.68	transcription factor	cell wall, homogalacturonan biosynthesis	[29]
AtPrx25|Peroxidase superfamily protein	AT2G41480	4.94	4.85	3.56	8.87	peroxidase	lignification, ROS generation	[30, 31]
RHD2,ATRBOHC,RBOHC|NADPH/respiratory burst oxidase protein D	AT5G51060	2.03	3.52	2.26	7.93	NADPH oxidase	ROS generation	[32, 33]
MYB77|myb domain protein 77	AT3G50060	7.71	5.81	2.89	1.91	transcription factor	auxin signaling, ROS metabolism	[34–36]
WRKY23,ATWRKY23|WRKYDNA-binding protein 23	AT2G47260	2.83	3.15	6.04	5.21	transcription factor	auxin flux, nematode response	[38–41]
Dof-type zinc finger DNA-binding family protein	AT4G24060	1.64	3.06	1.86	4.85	transcription factor	vascular patterning	[42]
ARR5,ATRR2,IBC6,RR5|response regulator 5	AT3G48100	3.48	2.90	3.83	5.95	histidine kinase	cytokinin signaling	[44–46]
DAG1|Dof-type zinc finger DNA-binding family protein	AT3G61850	2.42	3.33	2.18	2.57	transcription factor	phytohormone response	[47, 48]
DLO2|2-oxoglutarate(2OG) and Fe(II)-dependent oxygenase superfamily protein	AT4G10490	4.75	3.32	6.29	4.59	oxigenase	biotic stress response	[49]
Protein kinase superfamily protein, RIPK	AT2G05940	3.06	2.98	3.23	7.88	protein kinase	biotic stress response	[50]
bHLH25|Basic helix-loop-helix(bHLH) DNA-binding superfamily protein	AT4G37850	6.20	4.83	6.39	4.46	transcription factor	biotic stress response	[51]
WRKY48,ATWRKY48|WRKYDNA-binding protein 48	AT5G49520	1.32	3.39	4.16	4.46	transcription factor	biotic stress response	[52]
AtCYSTM4|CYSTEINE-RICH TRANSMEMBRANE MODULE 4	AT2G32190	8.91	2.36	3.89	3.73	transmembrane	abiotic stress response	[53]
AtMYB14|myb domain protein 14	AT2G31180	5.96	3.09	5.60	4.62	transcription factor	abiotic stress response	[54]
ATBAG7,BAG7|BCL-2-associated athanogene 7	AT5G62390	4.73	1.92	3.15	8.49	ER localization	abiotic stress response	[55–57]
HSF4,HSFB1,AT-HSFB1,ATHSF4|heatshock factor 4	AT4G36990	1.69	8.89	3.42	3.04	heat shock protein	abiotic- and biotic- stress responses	[58, 59]
BAM3|Leucine-richreceptor-like protein kinase family protein	AT4G20270	7.86	2.87	2.12	2.54	receptor kinase	abiotic stress response, development	[60–62]
HMG1,HMGR1,AtHMGR1|3-hydroxy-3-methylglutaryl CoA reductase1	AT1G76490	3.90	2.12	3.21	6.75	reductase	metabolic process	[63]
Galactosyltransferase family protein	AT1G77810	1.99	2.42	1.78	3.40	galactosyltransferase, Golgi apparatus localization	metabolic process	[64]
TUB1|tubulin beta-1chain	AT1G75780	6.23	4.14	2.23	2.87	tubulin	cytoskeleton	[65]
ATFD3,FD3|ferredoxin 3	AT2G27510	3.13	1.83	3.32	1.91	ferredoxin	Photosystem I	[66]
ANAC100,ATNAC5,NAC100|NAC domain containing protein 100	AT5G61430	2.58	3.53	3.66	4.75	transcription factor	miR164 target	[67]
C2calcium/lipid-binding plant phosphoribosyltransferase family protein MCTP16	AT5G17980	3.23	2.55	2.10	6.17	transmembrane	unknown	[68]
APK2B|proteinkinase2B	AT2G02800	1.58	2.29	7.11	8.23	Ser/Thr kinase	unknown	[69]
Adeninenucleotide alphahydrolases-like superfamily protein	AT3G17020	8.73	2.45	1.89	7.01	-	-	-
Plant invertase/pectin methylesterase inhibitor superfamily protein	AT5G62350	7.48	5.69	5.01	2.62	-	-	-
UNE1|Plant protein of unknown function (DUF641)	AT1G29300	3.50	3.15	3.05	4.89	-	-	-
GAD4|glutamate decarboxylase 4	AT2G02010	3.15	3.06	1.78	3.93	-	-	-
Protein of unknown function (DUF1635)	AT5G22930	2.64	4.18	2.64	2.87	-	-	-
RNA polymerase	AT5G56120	2.03	2.28	2.14	5.01	-	-	-
cotton fiber protein	AT3G60380	1.89	3.14	3.58	4.75	-	-	-
phosphatidylinositol 4-phosphate 5-kinase MSS4-like protein	AT1G29195	1.73	2.76	2.40	4.22	-	-	-
TIP41-like protein	AT3G54000	1.43	1.89	2.47	8.71	-	-	-

^a^ logFC value: the highest score among trinity contigs

Next, we categorize these candidate regulators based on their predicted biological and molecular functions, and discuss their contribution for gall development.

#### (1) Cell division and cytokinesis

In the gall, active cell division occurs to generate nutrient and shelter cells for insects, suggesting cell cycle regulation in the host tissue. We found several genes, involved in cell division and cytokinesis, that were up–regulated in four galls. The AtBRCA1 (At4g21070) is a direct transcriptional target of SUPPRESSOR OF GAMMA RESPONSE 1 (SOG1), and involved in DNA repair and cell cycle regulation [21–23]. FUSED Kinase (At1g50240) is involved in cytokinesis by interacting with kinesin protein in the phragmoplast [24–25]. Ethylene response factor 115 (ERF115/At5g07310) regulates the cell cycle of the quiescent center (QC) and surrounding stem cells in roots through direct transcriptional activation of *PHYTOSULFOKINE PRECURSOR 5* (*PSK5*) gene, which raises a sulfonated pentapeptide hormone molecule [26]. DOMINO1 (At5g62240) is a plant–specific gene family protein that is located in the nucleus and nucleolus, and is suggested to regulate nuclear size and cell division during embryogenesis [27]. Knockdown of dUTPase DUT1 (At3g46940) by RNAi causes DNA fragmentation and enhanced somatic homologous recombination [28], suggesting a DNA protection mechanism in galls. These up–regulated genes are likely to regulate cell proliferation in galls.

#### (2) Lignification and reactive oxygen species (ROS) generation

Lignification occurs in the cell layers surrounding the nutrient–rich cells, generating a shelter protecting larvae inside of the gall. AtTLP2 (At2g18280) is a transcription factor and regulates transcription of cell wall–related genes leading to homogalacturonan biosynthesis [29], suggesting that it is involved in biogenesis of cell wall components in the gall. AtPrx25 is a putative cationic cell–wall–bound peroxidase and is involved in lignin biosynthesis through oxidation of phenolic compounds and/or ROS generation [30–31]. These ROS are involved in many cellular processes including cell wall modification. Interestingly, ROOT HAIR DEFECTIVE 2 (RHD2, At5g51060), a NADPH oxidase that is involved in ROS production at the root hair tip, is up–regulated in the four galls, suggesting the involvement of ROS during gall development, possibly regulating cell wall structure for cell expansion and/or cellular signaling [32–33]. AtMYB77 (At3g50060) is a member of the R2R3–type transcription factor family and involved in metabolism of reactive oxygen species (ROS) by direct transcriptional regulation of the *ORBITALLY MANIFESTED GENE 1* (*OMG1*) [34]. These suggest that active ROS production is involved in lignification within the gall, generating a shelter–like structure.

#### (3) Phytohormone signaling and cell regeneration

Auxin is one of the key phytohormones in gall initiation and development. AtMYB77 is involved in lateral root formation via auxin signaling [35–36]. Since Arabidopsis cell regeneration mediates the process of lateral root development [37], the callus generation within the gall may be mediated by AtMYB77 and auxin signaling. The WRKY23 transcription factor is an auxin–response gene involved in embryogenesis and leaf venation patterning, through the regulation of PIN protein localization [38–40]. Overexpression of *WRKY23* affects the localization of PIN proteins, and also the leaf venation pattern [40]. Thus, up–regulation of WRKY23 can be involved in vascular patterning in galls through regulation of auxin flux. It is also activated at the site of nematode infection in roots [41], suggesting that WRKY23 also regulates biotic responses in the galls. DOF4.6 (At4g24060) is a member of plant–specific transcription factors, and expressed in vascular cells depending on auxin flux [42], suggesting its involvement in vascular development in galls.

Cytokinin is another key phytohormone in gall development, as well as other physiological functions in plants including cell division, cell regeneration and shoot differentiation [43]. Type–A Arabidopsis response regulator 5 (ARR5, At3g48100), a cytokinin primary response gene, is up–regulated in the four galls. ARR5 expression is activated by exogenous cytokinin and negatively regulates cytokinin signaling redundantly with the other ARRs, generating a feedback regulation to decrease sensitivity to cytokinin [44–46].

Dof AFFECTING GERMINATION 1 (DAG1)/At3g61850 controls hypocotyl cell elongation by affecting the expression of auxin–, ABA–and ethylene–related genes [47], as well as seed dormancy independently of ABA [48]. DAG1 is suggested to be involved in cellular morphogenesis through the regulation of phytohormone–related genes.

Together with previous studies, our results suggest that auxin and cytokinin are common regulators for gall development, and many responsive genes to these phytohormones are activated in galls. They seem to regulate cell proliferation and vascular differentiation during gall development.

#### (4) Biotic and abiotic stress responses

During gall initiation and development, insects may have to suppress the plant’s resistant system. Several genes involved in biotic–and abiotic–stress responses were up–regulated in galls. DLO2 (At4g10490), a homolog of DMR6 and acting redundantly with it, is upregulated in the four galls (Table 1). DLO2 is a negative regulator of plant defense and its overexpression results in reduced resistance to pathogens [49]. It is possible that insects regulate the expression of DLO2 and reducing plant defense. RIPK (At2g05940), a member of the receptor–like cytoplasmic kinase family, interacts directly with and phosphorylates RIN4, a negative regulator of immune responses against pathogen associated molecular pattern (PAMPs)–triggered immunity (PIT) [50]. RIKP overexpression lines are more susceptible to inoculation of *Pseudomonas syringae* DC3000, suggesting that up–regulation of RIPK in galls reduces the defense system in plants. bHLH25 (At4g37850), a putative transcription factor with a basic helix–loop–helix domain, is up–regulated in developing syncytia that are generated by invasion of cyst nematode *Heterodera schachtii* [51]. The *wrky48* mutant reduces the growth of the bacterial pathogen *P*. *syringae*, whereas overexpression leads to enhanced growth of the pathogen [52], suggesting that up–regulation of WRKY48 in galls represses the plant’s defense responses so that insects can survive.

Several abiotic–stress response genes are also up–regulated in the four galls. The expression of the cysteine–rich transmembrane module 4 (AtCYSTM4, At2g32190) is stimulated by salt, drought or oxidation stress [53]. AtMYB14 (At2g31180) is involved in cold tolerance [54]. The Bcl–2–associated athanogene (AtBAG7) is an ER–localized protein where it interacts with the molecular chaperon AtBiP2, and is involved in cold–, heat–and salinity–stress responses [55–56]. Sumoylated AtBAG7 interacts with WRKY29 in the nucleus where it is supposed to activate the molecular chaperon genes including AtBAG7 itself, leading to heat tolerance [57]. HsfB1 (At4g36690) encodes a heat shock protein that is suggested to be involved in thermotolerance response [58], as well as in salicylic acid–mediated resistance against pathogen challenge [59]. BAM3 (At4g20270) encodes a receptor–like kinase related to CLAVATA1 and functions as a receptor of CLAVATA3/EMBRYO SURROUNDING REGION (CLV3/ESR) peptides. So far, it is reported to be involved in suppression of root elongation and protophloem in roots as a receptor of CLE45 [60–61], and drought–stress response as a receptor of CLE25 [62]. CLE25 is up–regulated in galls of *E*. *japonica* and *G*. *obovatum* (Table 2; see below), suggesting that galls are responding to abiotic stresses, which are likely to be caused indirectly by insect infection.

**Table 2:** CLE, LRR-RLK, WOX, and MADS genes expressed in galls.

Gene symbol	AGI (*Arabidopsis thaliana*)	logFC^a^			
		*A*. *montana*	*E*. *japonica*	*G*. *obovatum*	*R*. *javanica*
**CLE**					
CLV3	AT2G27250	2.57			
CLE6	AT2G31085	6.05		7.34	
CLE7	AT2G31082		6.76		
CLE9	AT1G26600			1.62	
CLE25	AT3G28455		2.83	11.64	
CLE26	AT1G69970	1.32			3.80
CLE44	AT4G13195	3.75	2.87	2.07^b^	1.75
**LRR-RLK**					
	AT1G08590	2.84			4.15
RLK7	AT1G09970	1.69	3.11		6.07
	AT1G72180				
	AT1G75640	2.31		4.65	
CLV1	AT1G75820				2.29
	AT2G25790				7.74
ER	AT2G26330	2.64			7.94
	AT3G28040	3.40			
RLK5	AT4G28490	9.80		4.99	
	AT4G36180	5.65	3.03		
BRI1	AT4G39400			1.98	
	AT5G10020		2.42		2.75
FLS2	AT5G46330			5.00	9.80
	AT5G56040			3.07	
BAM1	AT5G65700	1.08	3.88		2.37
**WOX**					
WOX1	AT3G18010		7.02	4.75	
WOX2	AT5G59340				3.14
WOX4	AT1G46480	2.73	3.64		1.83
WOX13/HB-4	AT4G35550	1.40	2.66		
**MADS**					
AP1/AGL7	AT1G69120	12.13		8.85	5.83
AP3	AT3G54340			8.18	
PI	AT5G20240	2.49			
AG	AT4G18960	10.93		10.03	11.55
SEP1/AGL2	AT5G15800				12.39
SEP2/AGL4	AT3G02310	11.52			13.54
SEP3/AGL9	AT1G24260	7.29			11.41
SEP4/AGL3	AT2G03710	6.50			
SHP2/AGL5	AT2G42830			2.04	
SVP/AGL22	AT2G22540	3.65	3.69	9.23	
AGL62	AT5G60440		3.59		
TT16	AT5G23260				8.27
PHE	AT1G65330				7.23

^a^ logFC value: the highest score among trinity contigs.

^b^ *CLE44* in *G*. *obovatum* is up-regulated only in mature galls but not in young galls.

In summary, up–regulation of these abiotic–response genes suggests that in the gall, both biotic and abiotic stress responses are occurring during gall development.

#### (5) Metabolic processes

Plants biosynthesize secondary metabolites, such as terpene, phenolic acids, and alkaloids, and use them as a defense response. In the gall, the secondary metabolites are speculated to be biosynthesized and accumulated. 3–Hydroxy–3–methylglutaryl coenzyme A reductase (HMG1/HMGR, At1g76490) is involved in isoprenoid biosynthesis through regulation of ER morphogenesis [63]. At1g77810 encodes a member of the beta–(1,3)–galactosyltransferases, located in the Golgi apparatus [64]. This enzyme is involved in modification of arabinogalactan–proteins (AGPS), playing roles in various processes such as growth and development, programmed cell death, and signaling pathways [64]. Up–regulation of this gene in the gall may contribute to the biosynthesis of AGPs.

#### (6) Other up–regulated genes in the four galls

There are other up–regulated genes in the four galls: β–TUBULIN gene *TUB1* (At1g75780), a component of microtubules [65]; AtFD3 (At2g27510), a ferredoxin involved in photosystem I [66]. ANAC100 (At5t61430), a target of microRNA miR164 [67]; MCTP16 (At5g17980), encoding a multiple C2 domain and transmembrane region protein expressed in vascular tissue [68]; and APK2B (At2g02800), a serine/threonine protein kinase that is expressed in roots, leaves and flowers [69]. Future work will unveil their molecular and biological functions in galls.

### GO analysis suggests peptide signaling in galls

GO analysis predicts the biological and molecular functions of genes. We found that GO terms of peptide biosynthetic and peptide metabolic processes are common in four galls (S2 Table), as well as amide biosynthetic process and translation. Therefore, we extracted the CLV3/ESR–related (CLE) family genes from the gene list, and found that several genes are expressed in galls, especially *CLE44*, which is commonly up–regulated in the four galls (Table 2).

CLE peptides are small ligands that bind to the leucin–rich repeat receptor kinase family (LRR–RLK) CLV1/CLV2 proteins, and is involved in cell–cell communication during development, symbiosis, parasitism, and abiotic stress responses [70]. Several CLE and LRR-RLK genes are up–regulated in the galls (Table 2). CLE44 and CLE41 encoding the tracheary element differentiation inhibitory factor (TDIF) are involved in suppression of xylem cell differentiation in vascular stem cells [71]. Recent findings have shown that TDIF–like peptide from cyst nematodes can mimic the CLE function *in planta*, promoting vascular cell proliferation at the feeding site by activating the CLE and LRR–RLK pathway [72]. WOX4 is involved in promotion of vascular procambial and cambial stem cells depending on the CLE41/44 [73]. The *WOX4* gene as well as the other *WOX* family genes is up–regulated in several galls (Table 2), suggesting that CLE44 and WOX4 regulate the vascular generation in galls.

In many galls the vasculature is generated to connect to the source of host plant tissue, and this process is suggested to be regulated by CLE and LRR–RLK genes, together with the other factors such as the auxin–dependent process shown above. A previous study with grapevine gall has shown that *CLE44* and *WOX4* are up–regulated in galls [13], supporting our hypothesis that these factors are commonly involved in vascular development in galls.

### Genes involved in floral organ development

Shape and color of some galls show similarity to flowers and fruits. From the grapevine gall research, it is suggested that genes involved in reproductive organ development are up–regulated in developing galls [13]. Floral organ identity is determined by combined actions of the floral MADS genes [74–75]. We focused on MADS genes to find out if they are up–regulated in galls (Table 2). Interestingly many floral MADS genes were up–regulated in three plant galls, whereas they were not in the gall of *E*. *japonica*. This may be due to the different structure of galls: the gall of *E*. *japonica* is thinner than the other galls (Fig 1), suggesting less proliferation and differentiation of gall cells. This indicates that each gall mobilizes a distinct set of genes to generate each unique structure.

## Conclusions

Our results have provided a landscape of transcripts up–and down–regulated in four different galls, suggesting that galls are forced to mobilize the genes that are originally involved in other multiple biological processes to develop specific structure. The 38 commonly up–regulated genes may be involved in development of other leaf galls. Further transcriptome analyses of other plant species are required to validate this hypothesis. This work is based on the transcriptome of galls on plants and in order to understand the gall developmental mechanisms, we need to investigate the gall insects. To date, not many reports have been published except for that on the Hessian fly genome, transcriptome, and proteome (reviewed in [6]), and on *Schlechtendalia chinensis* [11]. Gall–causing insects, as well as the other galls on host plants, should be analyzed to understand the molecular mechanism of insect–plant interaction and gall development.

## Supporting information

S1 Fig Gene ontology (GO) analysis (biological process) of mature gall and leaf from *G*. *obovatum*. Colored dots indicate similar biological GO: blue, developmental process; red, phytohormone; and green, photosynthesis.
(TIF)

S1 Table RNA-sequencing analysis. (XLSX)

S2 Table GO analysis of genes up-regulated in galls. (XLSX)

## References

[1] Stone GN, Schönrogge K. The adaptive significance of insect gall morphology. Trends Ecol Evol 2003;18: 512–522. 10.1016/s0169-5347(03)00247-7

[2] Espírito–Santo MM, Fernandes GW. How many species of gall–inducing insects are there on earth, and where are they? Ann Entomol Soc Am. 2007;100: 95–99. 10.1603/0013-8746(2007)100[95:HMSOGI:2.0.CO;2

[3] Yamaguchi H, Tanaka H, Hasegawa M, Tokuda M, Asami T, Suzuki Y. Phytohormones and willow gall induction by a gall–inducing sawfly. New Phytol. 2012;196: 586–595. 10.1111/j.1469-8137.2012.04264.x 22913630

[4] Tanaka Y, Okada K, Asami T, Suzuki Y. Phytohormones in Japanese mugwort gall induction by a gall–inducing gall midge. Biosci Biotechnol Biochem. 2013;77: 1942–1948. 10.1271/bbb.130406 24018692

[5] Bartlett L, Connor EF. Exogenous phytohormones and the induction of plant galls by insects. Arthropod–Plant Interact. 2014;8: 339–348. 10.10007/s11829-011-9309-0

[6] Giron D, Huguet E, Stone GN, Body M. Insect–induced effects on plants and possible effectors used by galling and leaf–mining insects to manipulate their host–plant. J Insect Physiol. 2016;84: 70–89. 10.1016/j.jinsphys.2015.12.009 26723843

[7] Kaiser W, Huguet E, Casas J, Commin C, Giron D. Plant green–island phenotype induced by leaf–miners is mediated by bacterial symbionts. Proc R Soc B. 2010;277: 2311–2319. 10.1098/rspb.2010.0214 20356892 PMC2894905

[8] Body M, Kaiser W, Dubreuil G, Casas J, Giron D. Leaf–miners co–opt microorganisms to enhance their nutritional environment. J Chem Ecol 2013;39: 969–977. 10.1007/s10886-013-0307-y 23807431

[9] Barnewall EC, De Clerck–Floate RA. A preliminary histological investigation of gall induction in an unconventional galling system. Arthropod–Plant Interact. 2012;6: 449–459. 10.1007/s11829-012-9193-4

[10] Bailey S, Percy DM, Hefer CA, Cronk QCB. The transcriptional landscape of insect galls: psyllid (Hemiptera) gall formation in Hawaiian *Metrosideros polymorpha* (Myrtaceae). BMC Genomics. 2015;16: 943 10.1186/s12864-015-2109-9 26572921 PMC4647832

[11] Liu P, Yang ZX, Chen XM, Yang P. RNA–seq–based transcriptome and the reproduction–related genes for the aphid *Schlechtendalia chinensis* (Hemiptera, Aphididae). Genet Mol Res. 2017;16: gmr16019448 10.4238/gmr16019448 28340266

[12] Chen H, Liu J, Cui K, Lu Q, Wang C, Wu H, et al Molecular mechanisms of tannin accumulation in Rhus galls and genes involved in plant–insect interactions. Sci Rep. 2018;8: 9841 10.1038/s41598-018-28153-y 29959354 PMC6026138

[13] Schultz JC, Edger PP, Body MJA, Appel HM. A galling insect activates plant reproductive programs during gall development. Sci Rep. 2019;9: 1833 10.1038/s41598-018-38475-6 30755671 PMC6372598

[14] Guiguet A, Ohshima I, Takeda S, Laurans F, Lopez–Vaamonde C, Giron D. Origin of gall–inducing from leaf–mining in Caloptilia micromoths (Lepidoptera, Gracillariidae). Sci Rep. 2019;9: 6794 10.1038/s41598-019-43213-7 31043653 PMC6494848

[15] Guiguet A, Hamatani A, Amano T, Takeda S, Lopez–Vaamonde C, Giron D, et al Inside the horn of plenty: leaf–mining micromoth manipulates its host plant to obtain unending food provisioning. PLoS ONE. 2018;13: e0209485 10.1371/journal.pone.0209485 30576396 PMC6303051

[16] Brunner AM, Yakovlev IA, Strauss SH. Validating internal controls for quantitative plant gene expression studies. BMC Plant Biol. 2004;4: 1–7. 10.1186/1471-2229-4-1 15317655 PMC515301

[17] Grabherr MG, Haas BJ, Yassour M, Levin JZ, Thompson DA, Amit I, et al Full–length transcriptome assembly from RNA–seq data without a reference genome. Nat Biotechnol. 2011;29: 644–652. 10.1038/nbt.1883 21572440 PMC3571712

[18] Li H, Durbin R. Fast and accurate short read alignment with Burrows–Wheeler transform. Bioinformatics 2009;25: 1754–1760. 10.1093/bioinformatics/btp324 19451168 PMC2705234

[19] Robinson MD, McCarthy DJ, Smyth GK. EdgeR: a bioconductor package for differential expression analysis of digital gene expression data. Bioinformatics. 2010;26: 139–140. 10.1093/bioinformatics/btp616 19910308 PMC2796818

[20] Mi H, Muruganujan A, Ebert D, Huang X, Thomas PD. PANTHER version 14: more genomes, a new PANTHER GO–slim and improvements in enrichment analysis tools. Nucleic Acids Res. 2018;47: D419–D426. 10.1093/nar/gky1038 30407594 PMC6323939

[21] Lafarge S, Montané MH. Characterization of *Arabidopsis thaliana* ortholog of the human breast cancer susceptibility gene 1: AtBRCA1, strongly induced by gamma rays. Nucleic Acids Res. 2003;31: 1148–1155. 10.1093/nar/gkg202 12582233 PMC150221

[22] Sjogren CA, Bolaris SC, Larsen PB. Aluminum–dependent terminal differentiation of the Arabidopsis root tip is mediated through an ATR–, ALT2–, and SOG1–regulated transcriptional response. Plant Cell. 2015;27: 2501–2515. 10.1105/tpc.15.00172 26320227 PMC4815104

[23] Ogita N, Okushima Y, Tokizawa M, Yamamoto YY, Tanaka M, Seki M, et al Identifying the target genes of SUPPRESSOR OF GAMMA RESPONSE 1, a master transcription factor controlling DNA damage response in Arabidopsis. Plant J. 2018;94: 439–453. 10.1111/tpj.13866 29430765

[24] Oh SA, Johnson A, Smertenko A, Rahman D, Park SK, Hussey PJ, et al A divergent cellular role for the FUSED kinase family in the plant–specific cytokinetic phragmoplast. Curr Biol. 2005;15: 2107–2111. 10.1016/j.cub.2005.10.044 16332535

[25] Oh SA, Allen T, Kim GJ, Sidorova A, Borg M, Park SK, et al Arabidopsis Fused kinase and the Kinesin–12 subfamily constitute a signaling module required for phragmoplast expansion. Plant J. 2012;72: 308–319. 10.1111/j.1365-313X.2012.05077.x 22709276

[26] Heyman J, Cools T, Vandenbussche F, Heyndrickx KS, Leene JV, Vercauteren I, et al ERF115 controls root quiescent center cell division and stem cell replenishment. Science 2013;342: 860–863. 10.1126/science.1240667 24158907

[27] Lahmy S, Guilleminot J, Cheng CM, Bechtold N, Albert S, Pelletier G, et al DOMINO1, a member of a small plant–specific gene family, encodes a protein essential for nuclear and nucleolar functions. Plant J. 2004;39: 809–820. 10.1111/j.1365-313X.2004.02166.x 15341625

[28] Dubois E, Córdoba–Cañero D, Massot S, Siaud N, Gakière B, Domenichini S, et al Homologous recombination is stimulated by a decrease in dUTPase in Arabidopsis. PLoS ONE. 2011;6: e18658 10.1371/journal.pone.0018658 21541310 PMC3082524

[29] Wang M, Xu Z, Ahmed RI, Wang Y, Hu R, Zhou G, et al Tubby–like Protein 2 regulates homogalacturonan biosynthesis in Arabidopsis seed coat mucilage. Plant Mol Biol. 2019;99: 421–436. 10.1007/s11103-019-00827-9 30707395

[30] Shigeto J, Nagano M, Fujita K, Tsutsumi Y. Catalytic profile of Arabidopsis peroxidases, AtPrx–2, 25 and 71, contributing to stem lignification. PLoS ONE. 2014;9: e105332 10.1371/journal.pone.0105332 25137070 PMC4138150

[31] Shigeto J, Kiyonaga Y, Fujita K, Kondo R, Tsutsumi Y. Putative cationic cell–wall–bound peroxidase homologues in Arabidopsis, AtPrx2, AtPrx25, and AtPrx71, are involved in lignification. J Agri Food Chem. 2013;1: 3781–3788. 10.1021/jf400426g 23551275

[32] Foreman J, Demidchik V, Bothwell JHF, Mylona P, Miedema H, Torres MA, et al Reactive oxygen species produced by NADPH oxidase regulate plant cell growth. Nature 2003;22: 42–446. 10.1038/nature01485 12660786

[33] Takeda S, Gapper C, Kaya H, Bell E, Kuchitsu K, Dolan L. Local positive feedback regulation determines cell shape in root hair cells. Science 2008;19: 1241–1244. 10.1126/science.1152505 18309082

[34] Sng NJ, Kolaczkowski B, Ferl RJ, Paul AL. A member of the CONSTANS–like protein family is a putative regulator of reactive oxygen species homeostasis and spaceflight physiological adaptation. AoB Plants. 2018;11: ply075 10.1093/aobpla/ply075 30705745 PMC6348315

[35] Shin R, Burch AY, Huppert KA, Tiwari SB, Murphy AS, Guilfoyle TJ, et al The Arabidopsis transcription factor MYB77 modulates auxin signal transduction. Plant Cell. 2007;19: 2440–2453. 10.1105/tpc.107.050963 17675404 PMC2002618

[36] Zhao Y, Xing L, Wang X, Hou YJ, Gao J, Wang P, et al The ABA receptor PYL8 promotes lateral root growth by enhancing MYB77–dependent transcription of auxin–responsive genes. Sci Signal. 2015;7: ra53 10.1126/scisignal.2005051 24894996 PMC4298826

[37] Sugimoto K, Jiao Y, Meyerowitz EM. Arabidopsis regeneration from multiple tissues occurs via a root development pathway. Dev Cell. 2010;18: 463–471. 10.1016/j.devcel.2010.02.004 20230752

[38] Grunewald W, Smet ID, Lewis DR, Löfke C, Jansen L, Goeminne G, et al Transcription factor WRKY23 assists auxin distribution patterns during Arabidopsis root development through local control on flavonol biosynthesis. Proc Natl Acad Sci USA. 2012;109: 155–1559. 10.1073/pnas.1110541108 22307611 PMC3277162

[39] Grunewald W, Smet ID, Rybel BD, Robert HS, van de Cotte B, Willemesen V, et al Tightly controlled WRKY23 expression mediates Arabidopsis embryo development. EMBO Rep. 2013;14: 1136–1142. 10.1038/embor.2013.169 24157946 PMC3981095

[40] Prát T, Hajný J, Grunewald W, Vasileva M, Molnár G, Tejos R, et al WRKY23 is a component of the transcriptional network mediating auxin feedback on PIN polarity. PLoS Genet. 2018;14: e1007177 10.1371/journal.pgen.1007177 29377885 PMC5805370

[41] Grunewald W, Karimi M, Wieczorek K, de Cappelle EV, Wischnitzki E, Grundler F, et al A role for AtWRKY23 in feeding site establishment of plant–parasitic nematodes. Plant Physiol. 2008;148: 358–368. 10.1104/pp.108.119131 18599655 PMC2528098

[42] Gardiner J, Sherr I, Scarpella E. Expression of DOF genes identifies early stages of vascular development in Arabidopsis leaves. Int J Dev Biol 2010;54: 1389–1396, 10.1387/ijdb.093006jg 20563990

[43] Werner T, Schmülling T. Cytokinin action in plant development. Curr Opin Plant Biol. 2009;12: 527–538. 10.1016/j.pbi.2009.07.002 19740698

[44] D´Agostino IB, Deruère J, Kieber JJ. Characterization of the response of the Arabidopsis response regulator gene family to cytokinin. Plant Physiol. 2000;124: 1706–1717 10.1104/pp.124.4.1706 11115887 PMC59868

[45] Rashotte AM, Carson SDB, To JPC, Kieber JJ. Expression profiling of cytokinin action in Arabidopsis. Plant Physiol. 2003;132: 1998–2011. 10.1104/pp.103.021436 12913156 PMC181285

[46] To JPC, Haberer G, Ferreira FJ, Druère J, Mason MG, Schaller GE, et al Type–A Arabidopsis response regulators are partially redundant negative regulators of cytokinin signaling. Plant Cell 2004;16: 658–671. 10.1105/tpc.018978 14973166 PMC385279

[47] Lorrai R, Gandolfi F, Boccaccini A, Ruta V, Possenti M, Tramontano A, et al Genome–wide RNA–seq analysis indicates that the DAG1 transcription factor promotes hypocotyl elongation acting on ABA, ethylene and auxin signaling. Sci Rep. 2018;8: 15895 10.1038/s41598-018-34256-3 30367178 PMC6203721

[48] Papi M, Sabatini S, Bouchez D, Camilleri C, Costantino P, Vttorioso P. Identification and disruption of an Arabidopsis zinc finger gene controlling seed germination. Genes Dev. 2000;14: 28–33. 10640273 PMC316352

[49] Zeilmaker T, Ludwig NR, Elberse J, Seidl MF, Berke L, Doorn AV, et al DOWNY MILDEW RESISTANT 6 and DMR6–LIKE OXYGENASE 1 are partially redundant but distinct suppressors of immunity in Arabidopsis. Plant J. 2015;81: 210–222. 10.1111/tpj.12719 25376907

[50] Liu J, Elmore JM, Lin ZJD, Coaker G. A receptor–like cytoplasmic kinase phosphorylates the host target RIN4, leading to the activation of a plant innate immune receptor. Cell Host Microbe. 2011;9: 137–146. 10.1016/j.chom.2011.01.010 21320696 PMC3070605

[51] Jin J, Hewezi T, Baum TJ. The Arabidopsis bHLH25 and bHLH27 transcription factors contribute to susceptibility to the cyst nematode *Heterodera schachtii*. Plant J. 2011;65: 319–328. 10.1111/j.1365-313X.2010.04424.x 21223395

[52] Xing DH, Lai ZB, Zheng ZY, Vinod KM, Fan BF, Chen ZX. Stress–and pathogen–induced Arabidopsis WRKY48 is a transcriptional activator that represses plant basal defense. Mol Plant 2008;1: 459–470. 10.1093/mp/ssn020 19825553

[53] Xu Y, Yu Z, Zhang D, Huang J, Wu C, Yang G, et al CYSTM, a novel non–secreted cysteine–rich peptide family, involved in environmental stresses in *Arabidopsis thaliana*. Plant Cell Physiol. 2018;59: 423–438. 10.1093/pcp/pcx202 29272523

[54] Chen Y, Chen Z, Kang J, Kang D, Gu H, Qin G. AtMYB14 regulates cold tolerance in Arabidopsis. Plant Mol Biol Rep. 2013;31: 87–97. 10.1007/s11105-012-0481-z 24415840 PMC3881570

[55] Williams B, Kabbage M, Britt R, Dickman MB. AtBAG7, an Arabidopsis Bcl–2–associated athanogene, resides in the endoplasmic reticulum and is involved in the unfolded protein response. Proc Natl Acad Sci USA. 2010;107: 3088–6093. 10.1073/pnas.0912670107 20231441 PMC2851922

[56] Pan YJ, Liu L, Lin YC, Zu YG, Li LP, Tang ZH. Ethylene antagonizes salt–induced growth retardation and cell death process via transcriptional controlling of ethylene–, BAG–and senescence–associated genes in Arabidopsis. Frontiers Plant Sci. 2016;7: 696 10.3389/fpls.2016.00696 27242886 PMC4872043

[57] Li Y, Williams B, Dickman M. Arabidopsis B–cell lymphoma2 (Bcl–2)–associated athanogene 7 (BAG7)–mediated heat tolerance requires translocation, sumoylation and binding to WRKY29. New Phytol. 2017;214: 695–705. 10.1111/nph.14388 28032645

[58] Ikeda M, Mitsuda N, Ohme–Takagi M. Arabidopsis HsfB1 and HsfB2b act as repressors of the expression of heat–inducible Hsfs but positively regulate the acquired thermotolerance. Plant Physiol. 2011;157: 1243–1254. 10.1104/pp.111.179036 21908690 PMC3252156

[59] Pick T, Jaskiewicz M, Peterhänsel C, Conrath U. Heat shock factor HsfB1 primes gene transcription and systemic acquired resistance in Arabidopsis. Plant Physiol. 2012;159: 52–55. 10.1104/pp.111.191841 22427343 PMC3375984

[60] Depuydt S, Rodriguez–Villalon A, Santuari L, Wyser–Rmili C, Ragni L, Hardtke CS. Suppression of Arabidopsis protophloem differentiation and root meristem growth by CLE45 requires the receptor–like kinase BAM3. Proc Natl Acad Sci USA. 2013;110: 7074–7079. 10.1073/pnas.1222314110 23569225 PMC3637694

[61] Hazak O, Brandt B, Cattaneo P, Santiago J, Rodriguez–Villalon A, Hothorn M, et al Perception of root–active CLE peptide requires CORYNE function in the phloem vasculature. EMBO Rep. 2017;18: 1367–1381. 10.15252/embr.201643535 28607033 PMC5538625

[62] Takahashi F, Suzuki T, Osakabe Y, Betsuyaku S, Kondo Y, Dohmae N, et al A small peptide modulates stomatal control via abscisic acid in long–distance signaling. Nature 2018;556: 235–238. 10.1038/s41586-018-0009-2 29618812

[63] Ferrero S, Grados–Torrez RE, Leivar P, Antolín–Llovera M, López–Iglesias C, Cortadellas N. et al Proliferation and morphogenesis of the endoplasmic reticulum driven by the membrane domain of 3–hydroxy–3–methylglutaryl coenzyme A reductase in plant cells. Plant Physiol. 2015;168: 899–914. 10.1104/pp.15.00597 26015445 PMC4741317

[64] Qu Y, Egelund J, Gilson PR, Houghton F, Gleeson PA, Shultz CJ, et al Identification of a novel group of putative *Arabidopsis thaliana* ß–(1,3)–galactosyltransferases. Plant Mol Biol. 2008;68: 43–59. 10.1007/s11103-008-9351-3 18548197

[65] Chu B, Wilson TJ, McCune–Zierath C, Snustad DP, Carter JV. Two ß–tubulin genes, TUB1 and TUB8, of Arabidopsis exhibit largely nonoverlapping patterns of expression. Plant Mol Biol. 1998;37: 785–790. 10.1023/a:1006047129410 9678573

[66] Voss I, Goss T, Murozuka E, Altmann B, McLean KJ, Rigby SEJ, et al FdC1, a novel ferredoxin protein capable of alternative electron partitioning, increases in conditions of acceptor limitation at photosystem I J Biol Chem. 2011;286: 50–59. 10.1074/jbc.M110.161562 20966083 PMC3013009

[67] Sieber P, Wellmer F, Gheyselinck J, Riechmann JL, Meyerowitz EM. Redundancy and specialization among plant microRNAs: role of the MIR164 family in developmental robustness. Development. 2007;134: 1051–1060. 10.1242/dev.02817 17287247

[68] Liu L, Li C, Liang Z, Yu H. Characterization of multiple C2 domain and transmembrane region proteins in Arabidopsis. Plant Physiol. 2018;176: 2119–2132. 10.1104/pp.17.01144 29259105 PMC5841694

[69] Ito T, Takahashi N, Shimura Y, Okada K. A serine/threonine protein kinase gene isolated by an *in vivo* binding procedure using the Arabidopsis floral homeotic gene product, AGAMOUS. Plant Cell Physiol. 1997;38: 248–258. 10.1093/oxfordjournals.pcp.a029160 9150601

[70] Yamaguchi YL, Ishida T, Sawa S. CLE peptides and their signaling pathways in plant development. J Exp Bot. 2016;67: 4813–4826. 10.1093/jxb/erw208 27229733

[71] Hirakawa Y, Shinohara H, Kondo Y, Inoue A, Nakanomyo I, Ogawa M, et al Non–cell–autonomous control of vascular stem cell fate by a CLE peptide/receptor system. Proc Natl Acad Sci USA. 2008;105: 15208–15213, 10.1073/pnas.0808444105 18812507 PMC2567516

[72] Guo X, Wang J, Gardner M, Fukuda H, Kondo Y, Etchells JP, et al Identification of cyst nematode B–type peptides and modulation of the vascular stem cell pathway for feeding cell formation. PLoS Pathog. 2017;13: e1006142 10.1371/journal.ppat.1006142 28158306 PMC5319780

[73] Hirakawa Y, Kondo Y, Fukuda H. TDIF peptide signaling regulates vascular stem cell proliferation via the WOX4 homeobox gene in Arabidopsis. Plant Cell. 2010;22: 2618–2629. 10.1105/tpc.110.076083 20729381 PMC2947162

[74] Theissen G, Saedler H. Floral quartets. Nature. 2001;409: 469–471. 10.1038/35054172 11206529

[75] Krizek BA, Fletcher JC. Molecular mechanisms of flower development: an armchair guide. Nat Rev. 2005;6: 688–698. 10.1038/nrg1675 16151374

