# Supplementary figures and images for: Comparative transcriptome analysis of galls from four different host plants suggests the molecular mechanism of gall development
Source: PLoS One. 2019 Oct 24;14(10):e0223686. doi: 10.1371/journal.pone.0223686 (PMC6812778; doi:10.1371/journal.pone.0223686)

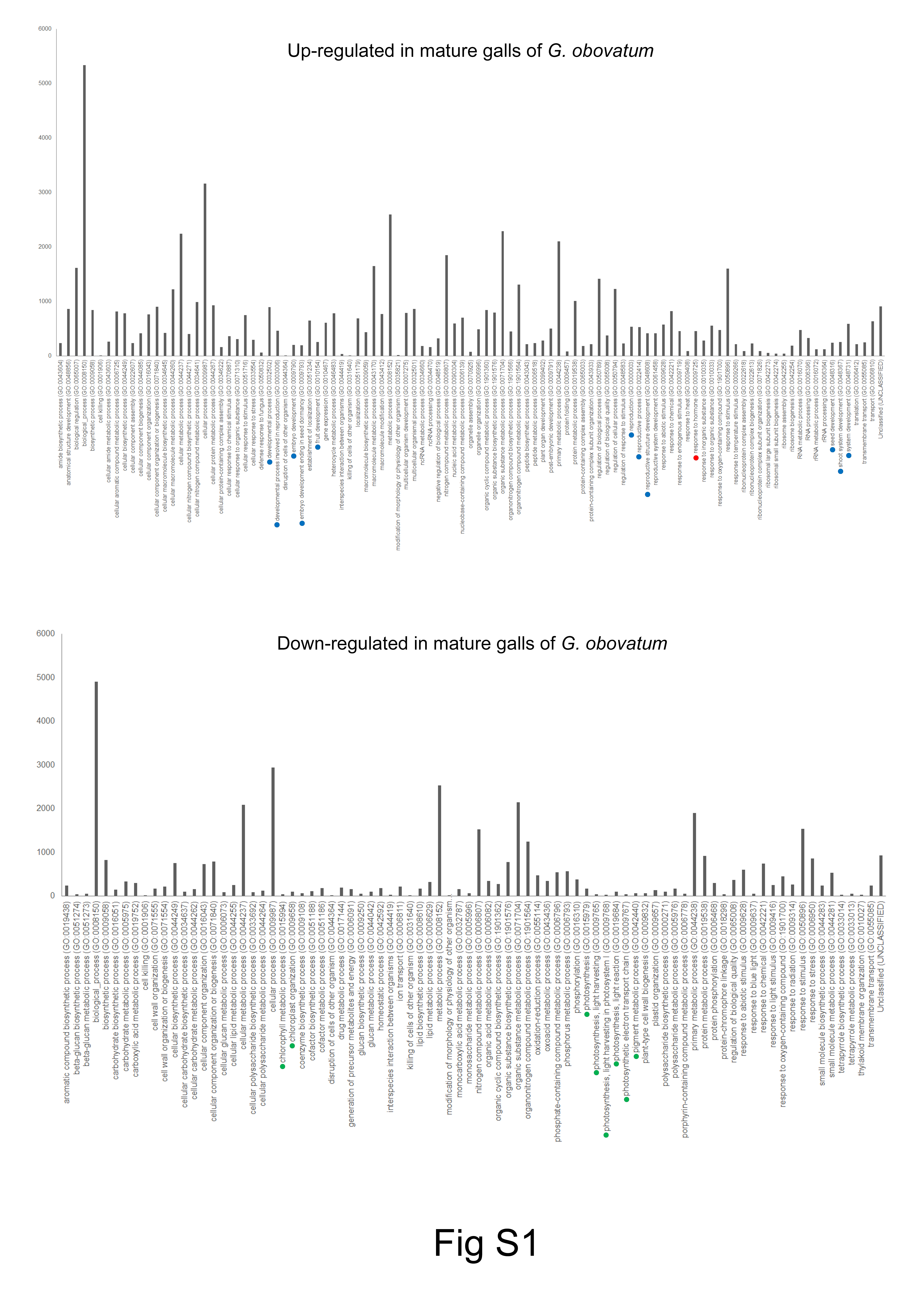

Supplement: S1 Fig — Colored dots indicate similar biological GO: blue, developmental process; red, phytohormone; and green, photosynthesis. (TIF) [file pone.0223686.s001.tif]
